# Tendência da Mortalidade por Doenças Cerebrovasculares no Brasil (1996-2015) e Associação com Desenvolvimento Humano e Vulnerabilidade Social

**DOI:** 10.36660/abc.20190532

**Published:** 2021-01-27

**Authors:** Carlos Dornels Freire de Souza, Denilson José de Oliveira, Leonardo Feitosa da Silva, Camila Damasceno dos Santos, Monaliza Coelho Pereira, João Paulo Silva de Paiva, Thiago Cavalcanti Leal, Renato de Souza Mariano, Amanda Karine Barros Ferreira de Araújo, Jussara Almeida de Oliveira Baggio

**Affiliations:** 1 Universidade Federal de Alagoas ArapiracaAL Brasil Universidade Federal de Alagoas - Campus Arapiraca, Arapiraca, AL - Brasil; 2 Faculdade São Francisco de Juazeiro JuazeiroBA Brasil Faculdade São Francisco de Juazeiro, Juazeiro, BA - Brasil; 3 Instituto de Medicina Integral Professor Fernando Figueira UPAE PetrolinaRE Brasil Instituto de Medicina Integral Professor Fernando Figueira – UPAE, Petrolina, RE - Brasil

**Keywords:** Encefalopatias/mortalidade, Epidemiologia, Desenvolvimento da Comunidade, Vulnerabilidade Social, Estudos de Séries Temporais, Morbimortalidade, Acidente Vascular Cerebral/mortalidade, Serviços Médicos de Emergência/organização e administração

## Abstract

**Fundamento:**

As doenças cerebrovasculares (DCBV) constituem a segunda causa de mortes no mundo.

**Objetivo:**

Analisar a tendência da mortalidade por DCVB no Brasil (1996-2015) e associação com o índice de desenvolvimento humano (IDH) e o índice de vulnerabilidade social (IVS).

**Métodos:**

Trata-se de estudo ecológico envolvendo as taxas de mortalidade padronizadas por DCBV. Os dados dos óbitos foram obtidos do Sistema de Informações sobre Mortalidade e os dados populacionais, do Instituto Brasileiro de Geografia e Estatística. Para as análises temporais, foi utilizado o modelo de regressão por pontos de inflexão, sendo calculado o percentual de variação anual ( *annual percent change* [APC]) e médio do período ( *average annual percent change* [AAPC]), com intervalo de confiança de 95% e significância de 5%. As tendências foram classificadas em crescente, decrescente ou estacionária. O modelo de regressão multivariada foi utilizado para testar a associação entre a mortalidade por DCBV, IDH e IVS.

**Resultados:**

Foram registrados 1.850.811 óbitos por DCBV no período estudado. Observou-se redução da taxa de mortalidade nacional (APC: -2,4; *p =* 0,001). Vinte unidades federativas apresentaram tendências significativas, sendo 13 de redução, incluindo todos das regiões Centro-Oeste (n = 4), Sudeste (n = 4) e Sul (n = 3). O IDH teve associação positiva e o IVS, associação negativa com a mortalidade (p = 0,046 e p = 0,026, respectivamente).

**Conclusão:**

O estudo mostrou comportamento epidemiológico desigual da mortalidade entre as regiões, sendo maior nos estados do Sudeste e Sul, porém com tendência significativa de redução, e menor nos estados do Norte e Nordeste, mas com tendência significativa de crescimento. O IDH e o IVS associaram-se com a mortalidade. (Arq Bras Cardiol. 2021; 116(1):89-99)

## Introdução

As doenças crônicas não transmissíveis (DCNT) têm ocupado lugar de destaque no cenário epidemiológico, representando atualmente o maior problema global de saúde, causando cerca de 38 milhões de mortes anualmente (70% de todos os óbitos), sendo 16 milhões delas consideradas prematuras (idade inferior a 70 anos).^1^ No Brasil, aproximadamente 75% dos óbitos são causados pelas DCNT, o que representa mais de 1 milhão de mortes a cada ano.^2^

O grupo das DCNT é composto por quatro subgrupos: cardiovasculares (DCV), câncer, doença respiratória crônica e diabetes melito. Dentre as DCV, destacam-se as doenças cerebrovasculares (DCBV), que figuram como a segunda causa de mortalidade no mundo, ficando atrás apenas das doenças isquêmicas do coração. Juntas, elas foram responsáveis por 15,2 milhões de mortes em 2016.^1 , 3^

Dentre os países da América Latina, o Brasil apresenta uma das maiores taxas de mortalidade por DCBV. Nas últimas décadas, houve um aumento expressivo do número de óbitos, passando de 104 mil, em 1990, para 144 mil, em 2015. Por outro lado, o país tem experimentado uma redução da taxa de mortalidade, sobretudo a precoce, que decresceu de 51,4% em 1990, para 35,1% em 2015.^4^

Considerando que o impacto das DCBV na morbimortalidade é um desafio para o desenvolvimento econômico e social das nações, sobretudo nos países em desenvolvimento, os quais concentram cerca de 80% de todos os óbitos,^1 , 5^ o monitoramento do comportamento temporal dos indicadores no Brasil, um país de dimensões continentais e com importantes desigualdades socioespaciais, é de fundamental importância para a definição de políticas públicas que possam impactar na situação de saúde da população.^6^

Nesse sentido, este trabalho objetivou analisar a tendência da mortalidade por DCVB no Brasil (1996-2015) e a associação com o índice de desenvolvimento humano (IDH) e o índice de vulnerabilidade social (IVS).

## Métodos

### Desenho do estudo, população e período

Trata-se de um estudo ecológico envolvendo todos os óbitos DCBV ocorridos no Brasil no período de 1996 a 2015 e o IDH e o IVS. Adotou-se como unidades de análise o Brasil, suas grandes regiões e as unidades da federação.

### Variáveis estudadas

Foram analisadas variáveis sociodemográficas: gêmero (masculino, feminino e ignorado), faixa etária em anos (0-4, 5-9, 10-14, 15-19, 20-29, 30-39, 40-49, 50-59, 60-69, 70-79, 80 e mais e idade ignorada), escolaridade em anos (nenhuma, 1-3, 4-7, 8-11, 12 ou mais e escolaridade ignorada) e estado civil (solteiro, casado, viúvo, separado, outro e estado civil ignorado). Para a análise de série temporal, foi incluída a variável taxa de mortalidade padronizada por idade e sexo em decorrência das DCBV. Para o componente de associação, foram selecionados dois índices sociais: i) o IDH e suas três dimensões (longevidade, educação e renda) e ii) o IVS e suas três dimensões (infraestrutura urbana, capital humano e renda e trabalho). Esses dois índices mensuram, respectivamente, o grau de desenvolvimento humano e o grau de vulnerabilidade social a que uma população está exposta.

### Fonte de dados e coleta

Os dados dos óbitos foram coletados do Sistema de Informações sobre Mortalidade (SIM) do Ministério da Saúde (http://datasus.saude.gov.br/).^7^ Considerou-se o Código Internacional de Doenças (CID-10) I60 a I69 – I60: hemorragia subaracnoide; I61: hemorragia intracerebral; I62: outras hemorragias intracranianas não traumáticas; I63: infarto cerebral; I64: acidente vascular cerebral (AVC) não especificado como hemorrágico ou isquêmico; I65: oclusão/estenose de artérias pré-cerebrais que não resultam em infarto cerebral; I66: oclusão/estenose de artérias cerebrais que não resultam em infarto cerebral; I67: outras doenças cerebrovasculares; I68: transtornos cerebrovasculares em doenças classificadas em outra parte; I69: sequelas de doenças cerebrovasculares.^8^ Os dados populacionais necessários ao cálculo dos indicadores foram obtidos do Instituto Brasileiro de Geografia e Estatística (IBGE).^9^

Para a obtenção das taxas, foram utilizadas as seguintes equações:

- Taxa de mortalidade anual: nº de óbitos por DCBV no local e ano/população no local e ano × 100.000 habitantes.
- Taxa de mortalidade do período (1996-2015): média de óbitos por DCBV da série temporal (1996-2015)/população do meio do período × 100.000.

Por fim, o IDH foi obtido do atlas de desenvolvimento humano (http://atlasbrasil.org.br/2013/) e o IVS, do atlas de vulnerabilidade social (http://ivs.ipea.gov.br/index.php/pt/), tendo como base o ano de 2010. Salienta-se que os dados de IDH e IVS somente são calculados nos anos censitários.

### Padronização das taxas de mortalidade

Para a redução dos efeitos da estrutura demográfica populacional, as taxas brutas foram padronizadas por gênero e idade pelo método direto, considerando-se como população padrão a brasileira do ano de 2010 (ano censitário) e as seguintes faixas etárias: 0-4, 5-9, 10-14, 15-19, 20-29, 30-39, 40-49, 50-59, 60-69, 70-79 e 80 ou mais.

### Tratamento estatístico

Para a análise temporal, foi utilizado o modelo de regressão por pontos de inflexão ( *joinpoint regression model* ). O modelo testa se uma linha com múltiplos segmentos é mais adequada para explicar o comportamento temporal de um conjunto de dados quando comparada com uma linha reta ou com menos segmentos. Desse modo, o *joinpoint* possibilita identificar a tendência de cada indicador (se estacionária, crescente ou decrescente), os pontos no tempo em que há modificação nessa tendência ( *joins* ), bem como a variação percentual anual ( *annual percent change* [APC]) e do período total ( *average annual percent change* [AAPC]).^10^ Na configuração do modelo, foram adotados os seguintes parâmetros: número mínimo de *joins* : zero; número máximo de *joins* : três; seleção do melhor modelo: teste de permutação de Monte Carlo (n = 4.499 permutações); método de autocorrelação dos erros: método baseado na data; intervalo de confiança: 95% (IC 95%); e nível de significância: 5%.

Para a análise de associação entre os indicadores sociais e a taxa padronizada de mortalidade, adotou-se o modelo de regressão multivariada ( *ordinary least square* [OLS]).

Para as análises, foram utilizados os *softwares* Joinpoint Regression 4.5.0.1 (National Cancer Institute, USA), GeoDa 1.10.0.8 (Universityof Illinois at Urbana- Champaign, USA) e QGis 2.14.11 (Open Source Geospatial Foundation, USA). As malhas territoriais necessárias para a confecção dos mapas foram provenientes do IBGE.

### Aspectos éticos

Por utilizar dados secundários de domínio público, nos quais não é possível a identificação dos sujeitos, dispensou-se a apreciação do Comitê de Ética em Pesquisa.

## Resultados

Entre 1996 e 2015, foram registrados 1.850.811 óbitos por DCBV no Brasil, expressando uma média de 92.540 casos/ano. Desse total, 50,68% (n = 938.044) ocorreram em indivíduos do gênero masculino e 77,80% (n = 1.440.170) em idosos. Ao estratificar segundo gênero, observou-se que a única faixa etária na qual a proporção de mulheres ultrapassou a de homens foi em indivíduos com idade igual ou superior a 80 anos. Destacou-se ainda a baixa escolaridade, uma vez que 39,94% (n = 739.233) eram analfabetos ou tinham até três anos de estudo. Nessa variável, observou-se uma elevada proporção de campos ignorados (38,29%/ n = 708.685) ( Tabela 1 ).

**Tabela 1:** – Caracterização sociodemográfica dos óbitos por DCBV, segundo gênero – Brasil (1996-2015)

Variáveis	Masculino n = 938.044 (50,68%)		Feminino n = 912.202 (49,29%)		Ignorado n = 565 (0,03%)		Total de Óbitos n = 1.850.811 (100%)	
	n	%	n	%	n	%	n	%
**Faixa etária**								
0-4	1.012	55,95	793	43,83	4	0,22	1.809	1,00
5-9	565	53,25	496	46,75	0	0,00	1.061	0,06
10-14	999	54,44	834	45,45	2	0,11	1.835	0,10
15-19	1.998	55,27	1.616	44,70	1	0,03	3.615	0,20
20-29	7.158	52,66	6.426	47,27	10	0,07	13.594	0,73
30-39	21.278	50,09	21.186	49,87	17	0,04	42.481	2,30
40-49	62.652	50,98	60.217	48,99	37	0,03	122.906	6,64
50-59	124.934	56,74	95.185	43,23	65	0,03	220.184	11,90
60-69	200.551	57,92	145.578	42,05	106	0,03	346.235	18,71
70-79	268.228	53,12	236.627	46,85	135	0,03	504.990	27,28
80 e mais	246.717	41,89	342.104	58,09	124	0,02	588.945	31,81
Idade ignorada	1.952	61,85	1.140	36,12	64	2,03	3.156	0,17
**Escolaridade**								
Nenhuma	162.163	42,91	215.672	57,07	90	0,02	377.925	20,42
1-3 anos	192.038	53,15	169.257	46,84	13	0,01	361.308	19,52
4-7 anos	126.285	53,86	108.156	46,13	11	0,01	234.452	12,67
8-11 anos	54.461	54,17	46.075	45,83	6	0,01	100.542	5,43
12 anos e mais	29.083	57,64	21.369	42,35	4	0,01	504.56	2,73
1-8 anos*	4.868	53,92	4.158	46,06	2	0,02	9.028	0,49
9-11 anos*	4.551	54,08	3.860	45,87	4	0,05	8.415	0,45
Ignorado	364.595	51,45	343.655	48,49	435	0,06	708.685	38,29
**Estado Civil**								
Solteiro	163.672	47,22	182.828	52,75	87	0,03	346.587	18,73
Casado	499.651	67,31	242.565	32,67	143	0,02	742.359	40,11
Viúvo	152.794	28,22	388.484	71,76	124	0,02	541.402	29,25
Separado	40.958	59,29	28.116	40,70	6	0,01	69.080	3,73
Outro	12.235	64,02	6.875	35,97	1	0,01	19.111	1,03
Ignorado	68.734	51,96	63.334	47,88	204	0,16	132.272	7,15

*Diferentes agrupamentos de escolaridade se devem às mudanças ocorridas no formulário de declaração de óbito (DO) em 2011.

**Figura 1:**
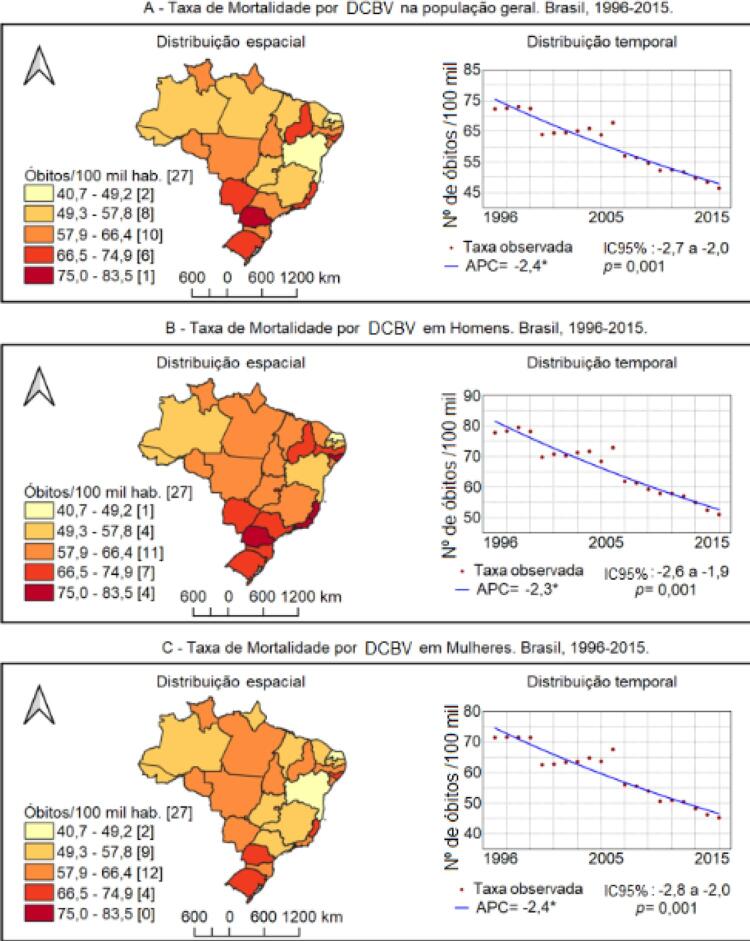
Distribuição espacial e tendência das taxas de mortalidade padronizada por DCBV nos estados brasileiros, geral e segundo gênero. Brasil (1996-2015). APC: Annual Percent Change; hab.: habitantes; nº: número; IC95%: intervalo de confiança de 95%; DCBV: doenças cerebrovasculares.

Ao longo da série temporal, a taxa de mortalidade por DCBV na população brasileira, considerando ambos os gêneros, apresentou tendência linear de redução (APC -2,4%; IC 95% -2,7 a -2,0; p = 0,001), passando de 72,3/100 mil (1996) para 46,4/100 mil (2015). Comportamentos semelhantes foram observados na população masculina (APC -2,3%; IC 95% -2,6 a -1,9; p = 0,001) e na feminina (APC -2,4%; IC 95% -2,8 a -2,0; p = 0,001), cujas taxas reduziram de 77,8 e 71,4/100 mil para 51,1 e 45,2/100 mil, respectivamente ( Figura 1 ).

Ainda conforme a Figura 1 , observa-se que a distribuição espacial das taxas médias é heterogênea, sendo maior nos estados das regiões Sudeste e Sul e menor no Norte. As maiores taxas médias gerais foram observadas nos estados do Paraná (75/100 mil) e Espírito Santo (71,3/100 mil), e as menores nos estados do Rio Grande do Norte (40,9/100 mil) e Bahia (48,0/100 mil). Esse mesmo cenário se repetiu para a mortalidade masculina (Paraná com 83,4/100 mil e Espírito Santo com 79,8/100 mil). Na população feminina, as maiores taxas foram observadas no Paraná (71,2/100 mil) e Rio Grande do Sul (69,2/100 mil) e as menores no Rio Grande do Norte (40,7/100 mil) e Bahia (49,1/100 mil).

Na etapa seguinte, analisou-se a tendência das taxas de mortalidade considerando toda a série temporal completa (1996-2015). A região Norte foi a única que apresentou tendência de crescimento da mortalidade na população geral (APC 0,4%; IC 95% 0,1 a 0,8; p < 0,001) e masculina (APC 0,7%; IC 95% 0,3 a 1,1; p < 0,001). As regiões Centro-Oeste, Sudeste e Sul apresentaram tendência decrescente, tanto na população geral quanto na masculina e feminina, com destaque para a região Sudeste com maior redução percentual na (APC 3,8%) ( Tabela 2 ).

**Tabela 2:** – Percentual de variação média anual (AAPC) das taxas de mortalidade padronizadas por DCBV, segundo gênero, no Brasil, regiões e unidades federadas (1996-2015)

Unidade espacial	Ambos os gêneros			Masculino			Feminino		
	Taxa^1^		AAPC (IC 95%) p valor	Taxa^1^		AAPC (IC 95%) p valor	Taxa^1^		AAPC (IC 95%) p valor
	1996	2015		1996	2015		1996	2015	
**Norte**	50,5	58,6	0,4* (0,1 a 0,8); p<0,001	51,0	61,6	0,7* (0,3 a 1,1); p<0,001	52,6	58,6	0,1 (-0,3 a 0,5); p=0,6
RO	68,2	51,4	-1,8* (-2,2 a -1,8); p<0,001	65,9	53,3	1,6* (-2,3 a -1,0); p<0,001	74,2	51,5	-1,9* (-2,3 a -1,4); p<0,001
AC	57,4	63,1	-0,1 (-1,9 a 1,7); p=0,9	59,5	62,7	0,4 (-2,3 a 3,1); p=0,8	58,7	66,9	0,4 (-4,3 a 5,4); p=0,9
AM	49,7	56,1	0,6* (0,2 a 1,1); p<0,001	49,7	58,0	0,9* (0,2 a 1,5); p<0,001	52,7	57,3	0,4 (-0,1 a 1,0); p=0,1
RR	75,5	46,8	-2,2* (-3,0 a -1,3); p<0,001	92,0	50,7	-2,0* (-3,6 a -0,4); p<0,001	56,4	44,2	-2,3* (-3,3 a -1,3); p<0,001
PA	46,3	61,8	1,2 (-0,8 a 3,4); p=0,2	45,7	66,0	1,9 (-0,3 a 4,1); p=0,1	49,5	60,8	0,1 (-2,0 a 2,2); p=0,9
AP	79,5	49,2	-1,7 (-7,8 a 4,9); p=0,6	77,1	54,3	-0,8 (-4,8 a 3,5); p=0,7	86,2	46,6	-1,2 (-3,8 a 1,5); p=0,4
TO	43,4	59,3	1,9* (0,9 a 2,9); p<0,001	48,0	60,1	1,4 (-1,3 a 4,3); p=0,3	40,0	60,7	2,3* (0,7 a 3,9); p<0,001
**Nordeste**	45,4	54,4	0,9 (-0,7 a 2,4); p=0,3	46,8	60,7	1,3 (-0,3 a 2,9); p=0,1	46,7	52,7	0,6 (-1,1 a 2,3); p=0,5
MA	29,0	68,2	4,6* (2,0 a 7,4); p<0,001	31,7	76,6	4,7* (2,5 a 7,0); p<0,001	27,2	64,8	4,3* (1,6 a 7,0); p<0,001
PI	33,3	76,9	3,9* (2,9 a 4,8); p<0,001	35,0	90,4	4,2* (2,9 a 5,5); p<0,001	33,0	70,3	4,0* (3,0 a 4,9); p<0,001
CE	42,0	55,1	1,3 (-0,2 a 2,8); p=0,1	43,5	62,6	1,7* (0,2 a 3,2); p<0,001	42,8	52,1	0,7 (-0,2 a 1,6); p=0,1
RN	33,0	38,0	0,9 (-0,1 a 1,8); p=0,1	34,4	43,7	1,3* (0,2 a 2,5); p<0,001	33,0	35,6	-0,4 (-0,8 a 1,5); p=0,5
PB	37,5	48,5	1,7* (0,4 a 3,0); p<0,001	39,0	52,2	1,9* (0,3 a 3,4); p<0,001	38,4	48,6	1,4 (-0,1 a 2,8); p=0,1
PE	64,8	58,0	-0,8 (-2,2 a 0,6); p=0,3	68,1	66,6	-0,4 (-1,0 a 0,2); p=0,2	65,9	55,2	-1,1 (-2,6 a 0,4); p=0,1
AL	55,5	69,3	0,8* (0,2 a 1,5); p<0,001	57,8	77,7	1,2* (0,6 a 1,8); p<0,001	57,1	66,9	0,5 (-0,5 a 1,5); p=0,3
SE	41,8	57,6	1,7* (1,0 a 2,3); p<0,001	45,7	64,7	1,9* (1,0 a 2,9); p<0,001	40,2	55,6	1,5* (0,5 a 2,5); p<0,001
BA	47,7	45,2	-0,0 (-0,6 a 0,5); p=0,9	47,4	47,5	0,2 (-0,3 a 0,8); p=0,4	51,0	46,2	-0,2 (-0,8 a 0,3); p=0,4
**Centro-Oeste**	69,5	46,3	-2,8* (-3,4 a -2,2); p<0,001	72,2	49,2	-2,7* (-3,3 a -2,2); p<0,001	69,1	46,2	-2,3 (-4,8 a 0,2); p=0,1
MS	76,9	52,9	-2,4* (-2,9 a -2,0); p<0,001	83,9	54,5	-2,4* (-3,0 a -1,8); p<0,001	73,1	54,5	-2,4* (-2,8 a 1,9); p<0,001
MT	65,7	44,2	-1,9* (-3,0 a -0,8); p<0,001	66,3	45,6	-2,1* (-3,5 a -0,6); p<0,001	67,6	44,8	-2,5* (-3,2 a -1,9); p<0,001
GO	64,2	46,2	-2,2* (-2,6 a -1,8); p<0,001	66,0	49,7	-1,6* (-2,7 a -0,4); p<0,001	65,1	45,6	-2,2 (-2,6 a -1,8); p<0,001
DF	81,3	41,6	-4,0* (-4,6 a -3,5); p<0,001	91,6	46,4	-4,0* (-4,6 a -3,4); p<0,001	77,2	40,5	-3,4 (-5,4 a -1,3); p<0,001
**Sudeste**	86,1	41,4	-3,8* (-4,1 a -3,4); p<0,001	96,3	45,8	-3,8* (-4,2 a -3,5); p<0,001	82,3	40,2	-3,8* (-4,2 a -3,4); p<0,001
MG	74,1	39,3	-3,2* (-3,5 a -2,9); p<0,001	81,0	41,5	-3,3* (-3,7 a -3,0); p<0,001	71,5	39,5	-3,0* (-3,3 a -2,8); p<0,001
ES	98,4	46,7	-3,6* (-4,3 a -2,9); p<0,001	108,7	51,7	-3,5* (-4,0 a -3,1); p<0,001	94,2	45,1	-3,5* (-4,2 a -2,8); p<0,001
RJ	101,6	42,3	-4,5* (-5,3 a -3,7); p<0,001	113,8	47,9	-4,3* (-4,9 a -3,7); p<0,001	97,7	40,7	-4,5* (-5,8 a -3,2); p<0,001
SP	84,3	41,7	-3,8* (-4,2 a -3,4); p<0,001	95,8	46,7	-3,8* (-4,0 a -3,5); p<0,001	79,4	40,1	-3,8* (-4,0 a -3,5); p<0,001
Sul	91,0	45,9	-3,7* (-4,1 a -3,2); p<0,001	96,9	49,5	-3,7* (-4,1 a -3,2); p<0,001	91,3	45,6	-3,6* (-4,0 a -3,2); p<0,001
PR	98,9	49,9	-3,8* (-4,1 a -3,6); p<0,001	108,0	55,3	-3,8* (-4,1 a -3,5); p<0,001	95,5	48,0	-3,8* (-4,1 a -3,5); p<0,001
SC	89,1	37,7	-4,4* (-4,8 a -4,0); p<0,001	94,4	39,7	-3,9* (-5,9 a -2,0); p<0,001	89,5	38,2	-4,3* (-4,7 a -3,9); p<0,001
RS	86,1	46,5	-3,1* (-4,2 a -1,9); p<0,001	88,9	49,4	-3,0* (-3,6 a -2,3); p<0,001	89,3	47,0	-3,4* (-4,1 a -2,6); p<0,001

*Significância estatística (p<0,05). 1Taxa de mortalidade/100 mil habitantes. AAPC: Average Annual Percent Change; RO: Rondônia; AC: Acre; AM: Amazonas; RR: Roraima; PA: Pará; AP: Amapá; TO: Tocantins; MA: Maranhão; PI: Piauí; CE: Ceará; RN: Rio Grande do Norte; PB: Paraíba; PE: Pernambuco; AL: Alagoas; SE: Sergipe; BA: Bahia; MG: Minas Gerais; ES: Espírito Santo; RJ: Rio de Janeiro; SP: São Paulo; PR: Paraná; SC: Santa Catarina; RS: Rio Grande do Sul; MS: Mato Grosso do Sul; MT: Mato Grosso; GO: Goiás; DF: Distrito Federal.

Na análise estratificada por unidade da federação, 20 estados apresentaram tendências significativas, sendo sete de crescimento e 13 de redução. Todos os estados das regiões Centro-Oeste, Sul e Sudeste apresentaram tendências decrescentes, com destaque para o Rio de Janeiro e Santa Catarina, com os maiores percentuais de redução. Por outro lado, dos sete estados com tendência de crescimento, cinco estão situados na região nordeste (Maranhão, Piauí, Paraíba, Alagoas e Sergipe) e dois no Norte (Amazonas e Tocantins) ( Tabela 2 ).

**Figura 2:**
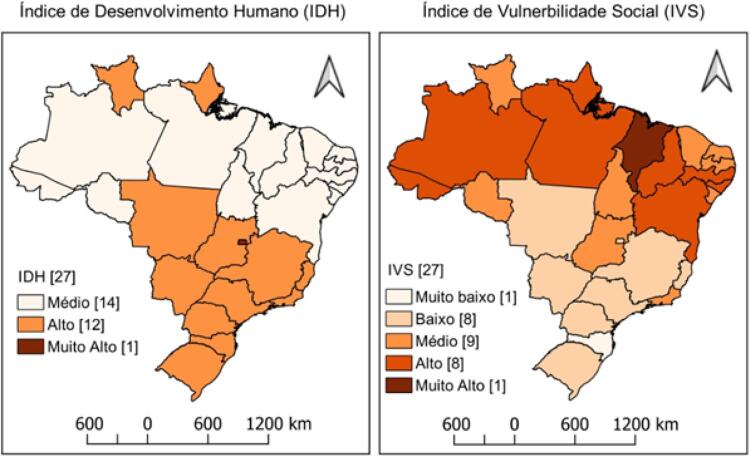
– Distribuição espacial do índice de desenvolvimento humano (IDH) e do índice de vulnerabilidade social (IVS) nos estados brasileiros. Brasil (2010).

Quanto ao IDH, apenas o Distrito Federal foi classificado como muito alto (IDH 0,824). Todos os estados do Nordeste e cinco do Norte apresentaram IDH médio (entre 0,600 e 0,699), destacando-se Alagoas e Maranhão com valores mais baixos (IDH 0,631 e 0,639, respectivamente). Em paralelo, esses mesmos estados das regiões Norte e Nordeste apresentaram os maiores valores no IVS, destacando-se o Maranhão com IVS muito alto (IVS 0,521). Todos os oito estados classificados como alta vulnerabilidade social estão situados nas regiões Norte (n = 4) e Nordeste (n = 4) ( Figura 2 ).

O modelo de regressão temporal mostrou ainda que os estados das regiões Norte e Nordeste foram os que apresentaram o maior número de segmentos na série temporal ( *joins* ), representando maior oscilação nas taxas ao longo dos anos. Ao analisar a mortalidade geral no Nordeste, por exemplo, quatro segmentos temporais foram encontrados: crescimento discreto (1996-2003), comportamento estacionário (2003-2006), tendência de redução (2006-2010) e novamente um comportamento estacionário (2010-2015). Dentre os estados dessa região, apenas a Bahia apresentou comportamento linear ( Tabela 3 ).

**Tabela 3:** – Percentual de variação anual das taxas de mortalidade padronizadas por DCBV, segundo gênero – Brasil (1996-2015)

Unidade espacial	Ambos os gêneros		Masculino		Feminino	
	Período	APC (IC 95%) p valor	Período	APC (IC 95%) p valor	Período	APC (IC 95%) p valor
**Norte**	1996-2015	0,4* (0,0 a 0,8); p<0,001	1996-2015	0,7* (0,3 a 1,1); p<0,001	1996-2015	0,1 (-0,3 a 0,5); p=0,6
RO	1996-2015	-1,8*(-2,2 a -1,8) ; p<0,001	1996-2015	1,6* (-2,3 a -1,0); p<0,001	1996-2015	-1,9* (-2,3 a -1,4); p<0,001
AC	1996-1999	-16,2 (-30,7 a 1,4); p=0,1	1996-2002	-5,6 *(-9,4 a -1,7); p<0,001	1996-1999	-16,2 (-30,7 a 1,4); p=0,1
	1999-2006	9,9* (3,1 a 17,2); p<0,001	2002-2006	13,2* (0,5 a 27,5); p<0,001	1999-2006	9,9* (3,1 a 17,2); p<0,001
	2006-2011	-6,4 (-17 a 5,6); p=0,2	2006-2015	-0,8 (-2,9 a 1,4); p=0,4	2006-2011	-6,4 (-17,0 a 5,6); p=0,2
	2011-2015	7,1 (-5,0 a 20,8); p=0,2			2011-2015	7,1 (-5,0 a 30,8); p=0,2
AM	1996-2015	0,6* (0,2 a 1,1); p<0,001	1996-2015	0,9* (0,2 a 1,5); p<0,001	1996-2015	0,4 (-0,1 a 1,0); p=0,1
RR	1996-2015	-2,2* (-3,0 a -1,3); p<0,001	1996-2015	-2,0* (-3,6 a -0,4); p<0,001	1996-2015	-2,3* (-3,3 a -1,3); p<0,001
PA	1996-2004	-0,8 (-2,9 a 1,2); p=0,9	1996-1998	11,6 (-1,1 a 25,9); p=0,1	1996-2004	-0,8 (-2,9 a 1,2); p=0,4
	2004-2008	6,3 (-3,3 a 16,8); p=1,4	1998-2001	-4,2 (-15,1 a 8,2); p=0,4		
	2008-2015	-2,3 (-4,7 a 0,2); p=-2,0	2001-2008	4,3* (2,2 a 6,4); p<0,001	2004-2008	6,3 (-3,3 a 16,8); p=0,2
			2008-2015	-0,5 (-2,1 a 1,2); p=0,5	2008-2015	-2,3 (-4,7 a 0,2); p=0,1
AP	1996-2007	-5,8* (-8,8 a -2,7); p=0,6	1996-2002	1,9 (-4,3 a 8,4); p=0,5	1996-2007	-5,8* (-8,8 a -2,7); p<0,001
	2007-2015	5,5* (0,1 a 11,3); p<0,001	2002-2006	-11,1 (-26,1 a 7,0); p=0,2	2007-2015	5,5* (0,1 a 11,3); p<0,001
			2006-2015	2,4 (-1,0 a 5,9); p=0,2		
TO	1996-2003	11,6* (7,5 a 15,8); p<0,001	1996-2000	3,1 (-2,5 a 8,9); p=0,3	1996-2003	11,6* (7,5 a 15,8); p<0,001
	2003-2015	-2,8* (-4,4 a -1,1); p<0,001	2000-2003	15,3 (-3,1 a 37,3); p=0,1	2003-2015	-2,8* (-4,4 a -1,1); p<0,001
			2003-2015	-2,3* (-3,3 a -1,3); p<0,001		
**Nordeste**	1996-2003	1,7* (0,5 a 2,9); p=0,3	1996-2003	2,1* (-0,9 a 3,3); p=0,1	1996-2003	1,4* (0,1 a 2,7); p<0,001
	2003-2006	7,4 (-1,5 a 17,2); p=1,9	2003-2006	6,6 (-2,3 a 16,4); p=0,1	2003-2006	8,3 (-1,5 a 19,1); p=0,1
	2006-2010	-4,5* (-8,5 a -0,2); p=0,3	2006-2010	-3,7 (-7,8 a 0,6); p=0,1	2006-2010	-5,1* (-9,5 a -0,5); p<0,001
	2010-2015	0,3 (-1,7 a 2,2); p=0,3	2010-2015	1,1 (-0,9 a 3,1); p=0,2	2010-2015	-0,4 (-2,5 a 1,8); p=0,7
MA	1996-2006	4,5* (2,2 a 6,9); p<0,001	1996-2000	0,1 (-7,1 a 7,8); p=1,0	1996-2003	4,5* (2,2 a 6,9); p<0,001
	2003-2006	18,3 (-0,2 a 40,2); p=0,1	2000-2007	13,4* (8,9 a 18,0); p<0,001	2003-2006	18,3 (-0,2 a 40,2); p=0,1
	2006-2015	-0,2 (-1,7 a 1,4); p=0,8	2007-2015	-0,1 (-2,6 a 2,6); p=1,0	2006-2015	-0,2 (-1,7 a 1,4); p=0,8
PI	1996-2006	8,9* (7,6 a 10,3); p<0,001	1996-2007	8,4* (6,8 a 10,1); p<0,001	1996-2006	8,9* (7,6 a 10,3); p<0,001
	2006-2015	-1,3 (-2,7 a 0,2); p=0,1	2007-2015	-1,4 (-3,7 a 1,1); p=0,2	2006-2015	-1,3 (-2,7 a 0,2); p=0,1
CE	1996-2007	2,8* (1,7 a 3,9); p<0,001	1996-1998	11,5 (-2,2 a 27,0); p=0,1	1996-2007	2,8* (1,7 a 3,9); p<0,001
	2007-2015	-2,2* (-3,9 a -0,4); p<0,001	1998-2008	2,0* (0,8 a 3,2); p<0,001	2007-2015	-2,2* (-3,9 a -0,4); p<0,001
			2008-2015	-1,2 (-2,9 a 0,5); p=0,1		
RN	1996-2009	2,4* (1,4 a 3,5); p<0,001	1996-2008	4,2* (2,9 a 5,4); p<0,001	1996-2009	2,4* (1,4 a 3,5); p<0,001
	2009-2015	-3,9* (-7,0 a -0,6); p<0,001	2008-2015	-3,3* (-5,9 a -0,6); p<0,001	2009-2015	-3,9* (-7,0 a -0,6); p<0,001
PB	1996-1998	-11,8* (-22,1 a 0,0); p<0,001	1996-1999	-5,5 (-12,3 a 2,3); p=0,1	1996-1998	-11,8* (-22,1 a 0,0); p<0,001
	1998-2007	8,9* (7,4 a 10,4); p<0,001	1999-2007	10,6* (8,3 A 13,0); p<0,001	1998-2007	8,9* (7,4 a 10,4); p<0,001
	2007-2015	-3,1* (-4,4 a -1,8); p<0,001	2007-2015	-3,5* (-5,1 a -1,8); p<0,001	2007-2015	-3,1* (-4,4 a -1,8); p<0,001
PE	1996-1998	5,1 (-2,8 a 13,7); p=0,2	1996-2006	1,1* (0,3 a 2,0); p<0,001	1996-1998	5,1 (-2,8 a 13,7); p=0,2
	1998-2001	-6,0 (-13,1 a 1,7); p=0,1	2006-2015	-2,0* (-3,0 a -1,1); p<0,001	1998-2001	-6,0 (-13,1 a 1,7); p=0,1
	2001-2005	4,9* (0,9 a 9,1); p<0,001			2001-2005	4,9* (0,9 a 9,1); p<0,001
	2005-2015	-3,2* (-3,8 a -2,6); p<0,001			2005-2015	-3,2* (-3,8 a -2,6); p<0,001
AL	1996-2007	2,4* (1,2 a 3,7); p<0,001	1996-2007	3,2* (2,4 a 4,0); p<0,001	1996-2007	2,4* (1,2 a 3,7); p<0,001
	2007-2015	-2,1 (-4,0 a -0,1); p<0,001	2007-2015	-1,5* (-2,7 a -0,3); p<0,001	2007-2015	-2,1* (-4,0 a -0,1); p<0,001
SE	1996-2005	5,7* (4,0 a 7,5); p<0,001	1996-2005	5,8* (4,1 a 7,4); p<0,001	1996-2005	5,7* (4,0 a 7,5); p<0,001
	2005-2015	-2,1* (-3,5 a -0,7); p<0,001	2005-2015	-1,4* (-2,7 a -0,1); p<0,001	2005-2015	-2,1* (-3,5 a -0,7); p<0,001
BA	1996-2015	-0,0 (-0,6 a 0,5); p=0,9	1996-2015	0,2 (-0,3 a 0,8); p=0,4	1996-2015	-0,2 (-0,8 a 0,3); p=0,4
**Centro-Oeste**	1996-2015	-2,8* (-3,4 a -2,2); p<0,001	1996-2015	-2,7* (-3,3 a -2,2); p<0,001	1996-2005	-0,5 (-2,0 a 1,0); p=0,5
					2005-2008	-0,9 (-23,1 a 7,7); p=0,2
					2008-2015	-1,6 (-3,8 a 0,6); p=0,1
MS	1996-2015	-2,4* (-2,9 a -2,0); p<0,001	1996-2015	-2,4* (-3,0 a -1,8); p<0,001	1996-2015	-2,4* (-2,8 a 1,9); p<0,001
MT	1996-2015	-1,9* (-3,0 a -0,8); p<0,001	1996-1998	9,7 (-3,5 a 24,6); p=0,1	1996-2015	-2,5* (-3,2 a -1,9); p<0,001
			1998-2010	-2,3* (-3,1 a -1,4); p<0,001		
			2010-2015	-5,9* (-8,6 a -3,2); p<0,001		
GO	1996-2015	-2,2* (-2,6 a -1,8); p<0,001	1996-1999	2,9 (-3,0 a 9,1); p<0,001	1996-2015	-2,2 (-2,6 a -1,8); p<0,001
			1999-2007	-3,8* (-5,3 a -2,3); p<0,001		
			2007-2015	-0,9 (-2,1 a 0,4); p=0,2		
DF	1996-1998	5,7 (-14,4 a 30,6); p=0,6	1996-2015	-4,0* (-4,6 a -3,4); p<0,001	1996-1998	5,7 (-14,4 a 30,6); p=0,6
	1998-2015	-4,4* (-5,1 a -3,7); p<0,001			1998-2015	-4,4* (-5,1 a -3,7); p<0,001
**Sudeste**	1996-2015	-3,8* (-4,1 a -3,4); p<0,001	1996-2015	-3,8* (-4,2 a -3,5); p<0,001	1996-2015	-3,8* (-4,2 a -3,4); p<0,001
MG	1996-2009	-2.6* (-3,2 a -1,9); p<0,001	1996-2015	-3,3* (-3,7 a -3,0); p<0,001	1996-2015	-3,0* (-3,3 a -2,8); p<0,001
	2009-2015	-5,5* (-7,4 a -3,5); p<0,001				
ES	1996-2015	-3,6 (-4,3 a -2,9); p<0,001	1996-2015	-3,5* (-4,0 a -3,1); p<0,001	1996-2009	-2,6* (-3,2 a -1,6); p<0,001
					2009-2015	-5,5* (-7,4 a -3,5); p<0,001
RJ	1996-2005	-5,1* (-5,9 a -4,4); p<0,001	1996-2010	-3,9* (-4,4 a -3,5); p<0,001	1996-2005	-5,1* (-5,9 a -4,4); p<0,001
	2005-2008	-0,6 (-8,9 a 8,4); p=0,9	2010-2015	-5,4* (-7,4 a -3,3); p<0,001	2005-2008	-0,6* (-8,9 a 8,4); p<0,001
	2008-2015	-5,4* (-6,5 a -4,3); p<0,001			2008-2015	-5,4* (-6,5 a -4,3); p<0,001
SP	1996-2015	-3,8* (-4,2 a -3,4); p<0,001	1996-2015	-3,8* (-4,0 a -3,5); p<0,001	1996-2015	-3,8* (-4,0 a -3,5); p<0,001
Sul	1996-2015	-3,7* (-4,1 a -3,2); p<0,001	1996-2015	-3,7* (-4,1 a -3,2); p<0,001	1996-2015	-3,6* (-4,0 a -3,2); p<0,001
PR	1996-2015	-3,8* (-4,1 a -3,6); p<0,001	1996-2015	-3,8* (-4,1 a -3,5); p<0,001	1996-2015	-3,8* (-4,1 a -3,5); p<0,001
SC	1996-2015	-4,4* (-4,8 a -4,0); p<0,001	1996-1998	5,2 (-9,2 a 21,9); p=0,5	1996-2015	-4,3* (-4,7 a -3,9); p<0,001
			1998-2002	-8,3* (-14,8 a -1,3); p<0,001		
			2002-2015	-3,9* (-4,7 a -3,2); p<0,001		
RS	1996-2012	-3,0* (-3,0 a -3,4); p<0,001	1996-1998	4,7 (-0,0 a 9,6); p<0,001	1996-2012	-3,0* (-3,4 a -2,6); p<0,001
	2012-2015	-5,4* (-10,0 a -0,6); p<0,001	1998-2006	-3,9* (-4,5 a -3,3); p<0,001		
			2006-2010	-1,6 (-3,8 a 0,7); p=0,1	2012-2015	-5,4* (-10,0 a -0,6); p<0,001
			2010-2015	-5,5* (-6,4 a -4,5); p<0,001		

*Significância estatística (p<0,05). APC: Annual Percent Change; RO: Rondônia; AC: Acre; AM: Amazonas; RR: Roraima; PA: Pará; AP: Amapá; TO: Tocantins; MA: Maranhão; PI: Piauí; CE: Ceará; RN: Rio Grande do Norte; PB: Paraíba; PE: Pernambuco; AL: Alagoas; SE: Sergipe; BA: Bahia; MG: Minas Gerais; ES: Espírito Santo; RJ: Rio de Janeiro; SP: São Paulo; PR: Paraná; SC: Santa Catarina; RS: Rio Grande do Sul; MS: Mato Grosso do Sul; MT: Mato Grosso; GO: Goiás; e DF: Distrito Federal.

Por fim, o modelo de regressão mostrou associação positiva entre a taxa média de mortalidade e o IDH municipal (p = 0,046) e sua dimensão renda (p = 0,029), e associação negativa com o IVS (p = 0,026) e em duas dimensões: capital humano (p = 0,046) e renda e trabalho (p = 0,018) ( Tabela 4 ).

**Tabela 4:** – Modelo de regressão (ordinay least square [OLS]) entre a taxa de mortalidade por DCBV e o índice de desenvolvimento humano e índice de vulnerabilidade social – Brasil (1996-2015)

Variável	Coeficiente	Estatística t	p valor
**Índice de desenvolvimento humano municipal (IDHM)**	61,588	2,091	0,046*
IDHM longevidade	90,265	1,866	0,073
IDHM educação	47,075	1,861	0,074
IDHM renda	56,476	2,301	0,029*
**Índice de vulnerabilidade social (IVS)**	-40,802	-2,353	0,026*
IVS infraestrutura urbana	-15,998	-1,110	0,277
IVS capital humano	-31,883	-2,092	0,046*
IVS renda e trabalho	-35,322	-2,528	0,018*

*Associação significativa.

## Discussão

O Brasil apresenta uma das maiores taxas de mortalidade por DCBV dentre os países da América Latina e ainda muito superior ao de nações desenvolvidas.^11^ Todavia, um comportamento temporal de declínio da taxa nacional tem sido observado ao longo das últimas décadas,^1^ tanto na população masculina quanto na feminina, corroborando a literatura existente nacional e internacional.^12 - 15^

Muitos autores têm destacado que tal redução da mortalidade pode ser explicada pela ampliação do acesso aos serviços de saúde e da adoção de estratégias de prevenção.^14 , 15^ No Brasil, destaca-se a implantação da atenção primária à saúde (APS), que, por meio da Estratégia Saúde da Família (ESF) desenvolve ações de controle de fatores de risco, a exemplo do incentivo à prática de atividade física e da adoção de hábitos alimentares salutares, programa de controle do tabagismo, diagnóstico e acompanhamento sistemático das condições crônicas (p. ex., hipertensão e diabetes) e acesso à assistência farmacêutica.^16 , 17^ Entre 1998 e 2017, houve uma intensa expansão do número de equipe de saúde da família, passando de pouco mais de 2 mil para 41 mil, alcançando uma cobertura de 70% da população brasileira, o que corresponde a aproximadamente 143 milhões de pessoas.^17 , 18^ Estudos têm mostrado associação entre a expansão da atenção primária e a redução da mortalidade por doenças como infarto agudo do miocárdio e doenças cerebrovasculares.^19^

Além da APS, o Brasil também avançou consideravelmente no atendimento ao paciente com DCBV. Em 1997, foi implantada a primeira unidade de AVC no Brasil, situada em Joinville-SC. A partir dessa experiência, em 2008, o Ministério da Saúde iniciou a organização da rede nacional de atendimento ao AVC, resultando na promulgação da portaria nº 665/2012, com a finalidade de implantar serviços de referência em todo o país.^20 , 21^

Outra ação importante diz respeito ao Plano de Ações Estratégicas para o Enfrentamento das DCNT. Implantado em 2011 pelo Ministério da Saúde, o plano estabeleceu um conjunto de metas para o país, como a redução da mortalidade prematura por DCNT, da prevalência de tabagismo e do consumo de álcool na população, aumento da prevalência da prática de atividade física e do consumo de frutas e contenção do crescimento da obesidade.^22^

Na análise regional, evidenciamos um comportamento heterogêneo no padrão de mortalidade pelas DCBV no país, corroborando outros estudos.^4 , 23^ As taxas de mortalidades foram maiores nas regiões Sudeste e Sul, porém com tendência de redução significativa. Em contraste, o Norte e o Nordeste apresentaram as menores taxas, mas com tendência de crescimento significativo ao longo da série histórica. Esse contexto epidemiológico-espacial heterogêneo é resultado das diferenças sociais, econômicas, demográficas e epidemiológicas existentes entre as regiões. Em virtude disso, os achados devem ser analisados sob a ótica de três dimensões: i) transição demográfica e epidemiológica; ii) determinantes sociais da saúde; e iii) qualidade dos sistemas de informações.

Desde os anos 1940, o Brasil tem passado por importantes transformações demográficas: redução da taxa de mortalidade geral e o declínio da natalidade resultaram em grandes mudanças no regime demográfico e na estrutura etária da população, com significativo aumento de idosos.^24^ Em 2000, essa população era de pouco mais de 14,2 milhões, passando para 19,6 milhões em 2010, devendo alcançar 41,5 milhões já em 2030,^25^ com maior concentração das regiões Sudeste e Sul. O impacto do processo de envelhecimento populacional no padrão de morbimortalidade é significativo, uma vez que implica aumento das doenças crônicas,^26^ dentre as quais se destacam as DCBV. Em nosso estudo, 77,8% dos óbitos ocorreram em idosos.

Estudos apontam que o risco de mortalidade por DCBV na população idosa é substancialmente maior do que em outras faixas etárias. Uma das razões é o acúmulo de fatores de risco, como hipertensão, diabetes, dislipidemias, etilismo, tabagismo e hábitos alimentares inadequados.^27 , 28^ Em nível nacional, por exemplo, a prevalência de hipertensão pode acometer 68% dos idosos.^29^

Ademais, o processo de transição demográfica ocorre em concomitância com uma segunda transição, a epidemiológica, caracterizada pelas mudanças no perfil de adoecimento da população.^30^ Nas últimas décadas, tem sido observado declínio das doenças infecciosas e parasitárias e aumento da ocorrência das crônico-degenerativas, muitas das quais, conforme mencionado anteriormente, elevam o risco de mortalidade por DCBV.^27^ As regiões Norte e Nordeste são as mais expostas à vulnerabilidade social e ao menor desenvolvimento humano, resultando em maior mortalidade por doenças decorrentes desse contexto social desfavorável e menor participação das DCBV, diferentemente do que se observa nas regiões mais desenvolvidas do país (Sudeste e Sul). Nesse sentido, as maiores taxas observadas nos estados mais desenvolvidos refletem as diferenças sociais e, consequentemente, maior participação das condições crônicas no perfil de mortalidade. Em contrapartida, regiões mais vulneráveis podem apresentar menores taxas em razão da persistência da mortalidade em decorrência de doenças relacionadas à pobreza.^32^

As duas transições não ocorrem de modo homogêneo no Brasil, havendo um descompasso entre as regiões.^31^ A transição desigual explica, em partes, as diferenças entre as regiões brasileiras no tocante à mortalidade por DCBV. Esse cenário justifica a associação positiva entre a mortalidade por DCBV e o desenvolvimento humano e a associação negativa desta com a vulnerabilidade social, o que representa a influência do contexto epidemiológico e social no perfil de mortalidade da população.

Todavia, a análise isolada das taxas não é suficiente para compreender a dinâmica epidemiológica das DCBV, sendo necessário refletir sobre a sua tendência ao longo da série temporal. Nas regiões Norte e Nordeste, em geral, as taxas mostraram padrão temporal de crescimento e, nas regiões Sudeste e Sul, observou-se declínio. Esses achados refletem a influência dos determinantes sociais da saúde no padrão de mortalidade por DCBV. As condições socioeconômicas, incluindo desenvolvimento humano, condição de renda e situação educacional, exercem influência significativa no risco de um indivíduo morrer por esse grupo de doenças.^4 , 6 , 33 - 35^

Recente estudo da Carga Global de Doenças ( *Global Burden of Disease* , 2015) mostrou que os estados brasileiros situados no tercil mais baixo do índice de desenvolvimento social apresentaram reduções menores das taxas de mortalidade, quando comparados aos estados situados no tercil superior de desenvolvimento.^4^ Nesse mesmo estudo, o tercil inferior foi composto, unicamente, por estados das regiões Norte e Nordeste. Sugere-se, portanto, que as melhores condições de vida apresentam dupla influência na tendência da mortalidade: i) reduzem os fatores de risco para a ocorrência dos eventos de doença; e ii) contribuem para a sobrevida dos pacientes quando tais eventos ocorrem, reduzindo a chance de ocorrência do óbito.

Por fim, é necessário refletir ainda sobre a qualidade dos registros de mortalidade, sendo este um desafio para o adequado monitoramento das condições de saúde da população. O inadequado preenchimento da declaração de óbito, resultando em elevado número de códigos *garbage,* as dificuldades em realizar investigação epidemiológica dos óbitos registrados com causa mal definida e a falta de recursos humanos capacitados para atuação nos serviços de vigilância do óbito são problemas comuns evidenciados em todo o país, embora as regiões Norte e Nordeste sejam as mais atingidas pelo problema.^36 , 37^ A qualidade duvidosa das informações configura-se como uma importante limitação deste estudo.

Entre 1996 e 2005, o percentual de óbitos com causas mal definidas nessas regiões foi superior a 20%, sendo ainda maior na população idosa quando comparada com outras faixas etárias.^38^ Nesse sentido, as taxas de mortalidade no Norte e Nordeste, por exemplo, podem ser superiores àquelas que evidenciamos neste estudo. Por outro lado, é necessário destacar que, nos últimos anos, importantes avanços na qualidade da informação foram observados nessas regiões.^13^

## Conclusão

Conclui-se que a mortalidade por DCBV no Brasil apresenta comportamento epidemiológico desigual entre as regiões. As maiores taxas foram observadas nos estados com melhor grau de desenvolvimento humano e menor vulnerabilidade social, mas com tendência decrescente ao longo da série temporal. Por outro lado, nos estados com menor desenvolvimento e maior vulnerabilidade, as taxas foram menores, mas com tendência de crescimento. Nesse sentido, advogamos que as políticas públicas devem ser desenvolvidas considerando o contexto regional/local.
