# Supplementary material for: Tendência da Mortalidade por Doenças Cerebrovasculares no Brasil (1996-2015) e Associação com Desenvolvimento Humano e Vulnerabilidade Social
Source: Arq Bras Cardiol. 2021 Jan 27;116(1):89–99. [Article in Portuguese] doi: 10.36660/abc.20190532 (PMC8159516; doi:10.36660/abc.20190532)
Supplement: Translation

# Translation: Cerebrovascular Disease Mortality Trend in Brazil (1996 To 2015) and Association with Human Development Index and Social Vulnerability

**Authors:** Carlos Dornels Freire de Souza, Denilson José de Oliveira, Leonardo Feitosa da Silva, Camila Damasceno dos Santos, Monaliza Coelho Pereira, João Paulo Silva de Paiva, Thiago Cavalcanti Leal, Renato de Souza Mariano, Amanda Karine Barros Ferreira de Araújo, Jussara Almeida de Oliveira Baggio

**Affiliations:** 1 Brazil Universidade Federal de Alagoas - Campus Arapiraca, Arapiraca, AL - Brazil; 2 Brazil Faculdade São Francisco de Juazeiro, Juazeiro, BA - Brazil; 3 Brazil Instituto de Medicina Integral Professor Fernando Figueira – UPAE, Petrolina, RE - Brazil

**Keywords:** Brain Diseases/mortality, Epidemiology, Community Development, Social Vulnerability, Time Seies Studies, Morbimortality, Stroke/mortality, Emergency Medical Emergencies/organization and administration

## Abstract

**Background:**

Cerebrovascular diseases (CBVD) are the second major cause of death in the world.

**Objective:**

To analyze the mortality trend of CBVD in Brazil (1996 to 2015) and its association with Human Development Index (HDI) and the Social Vulnerability Index (SVI).

**Methods:**

This is an ecological study. We analyzed the mortality rate standardized by CBVD. Death data were obtained from the Mortality Information System (SIM) and populational data from the Brazilian Institute of Geography and Statistics (IBGE). The model of regression by inflection points (Joinpoint regression) was used to perform the temporal analysis, calculating the Annual Percent Change (APC) and Average Annual Percent Change (AAPC), with 95% of confidence interval and a significance of 5%. Trends were classified as increasing, decreasing or stationary. A multivariate regression model was used to analyze the association between mortality by CBVD, HDI and SVI.

**Results:**

During this period, 1,850,811 deaths by CBVD were recorded. We observed a reduction in the national mortality rate (APC -2.4; p = 0.001). Twenty federation units showed a significant trend, of which 13 showed reduction, including all states in the Midwest (n=4), Southeast (n=4) and South (n=3). The HDI was positively associated and the SVI was negatively associated with mortality (p = 0.046 and p = 0.026, respectively).

**Conclusion:**

An unequal epidemiological course of mortality was observed between the regions, being higher in the Southeast and South states, with a significative tendency of reduction, and lower in the North and Northeast states, but with a significative tendency of increase. HDI and SVI showed an association with mortality. (Arq Bras Cardiol. 2021; 116(1):89-99)

## Introduction

Chronic non-communicable diseases (CNCD) have occupied a prominent place in the epidemiological scenario, representing the biggest global health problem and causing about 38 million deaths annually (70% of all deaths), 16 million of which are considered premature (age < 70 years).^1^In Brazil, approximately 75% of deaths are caused by CNCD, which represents more than 1 million deaths each year.^2^

The CNCD group consists of four subgroups: cardiovascular diseases (CVD), cancer, chronic respiratory disease and diabetes mellitus. Among the CVD, cerebrovascular diseases (CBVD) stand out, being the second leading cause of mortality in the world, behind ischemic heart diseases. Together, they were responsible for 15.2 million deaths in 2016.^1 , 3^

Of the Latin American countries, Brazil has one of the highest mortality rates due to CBVD. In the last decades, there was a significant increase in the number of deaths, from 104,000 in 1990 to 144,000 in 2015. On the other hand, the country has experienced a reduction in mortality rates, especially regarding early mortality, which decreased from 51.4% in 1990 to 35.1% in 2015.^4^

The impact of CBVD on morbidity and mortality is a challenge for the economic and social development of nations, especially in developing countries, which concentrate about 80% of all CBVD deaths.^1 , 5^Monitoring the temporal behavior of indicators in Brazil, a country of continental dimensions and with important socio-spatial inequalities, is of fundamental importance for the definition of public policies that can impact the population’s health situation.^6^

In this sense, this study aimed to analyze the trend of mortality from CBVD in Brazil (1996-2015) and its association with the Human Development Index (HDI) and the Social Vulnerability Index (SVI).

## Methods

### Study design, population and period

This is an ecological study involving all CBVD deaths that occurred in Brazil from 1996 to 2015 and the HDI and SVI. The entire country, the country regions and the federation units were adopted as the analysis units.

### Variables

We analyzed the following sociodemographic variables: gender (male, female and unknown), age groups – in years (0-4, 5-9, 10-14, 15-19, 20-29, 30-39, 40-49, 50- 59, 60-69, 70-79, 80 and over and unknown age), education - in years (illiterate, 1-3, 4-7, 8-11, 12 or more and unknown education) and marital status (single, married, widowed, divorced, other and unknown marital status). For the time series analysis, the variable mortality rate standardized by age and gender due to CBVD was included. For the association component, two social indices were selected: i) the HDI and its three dimensions (longevity, education and income) and ii) SVI and its three dimensions (urban infrastructure, human capital and income and work). These two indices measure, respectively, the degree of human development and the degree of social vulnerability to which a population is exposed.

### Data source and data collection

Death data were collected from the Ministry of Health’s Mortality Information System (SIM) (http://datasus.saude.gov.br/).^7^The International Disease Code (ICD-10) I60 to I69 was considered: I60- Subarachnoid hemorrhage; I61- Intracerebral hemorrhage; I62- other non-traumatic intracranial hemorrhages; I63- Cerebral infarction; I64- Stroke not specified as hemorrhagic or ischemic; I65- Occlusion / stenosis of pre-cerebral arteries that do not result in cerebral infarction; I66- Occlusion / stenosis of cerebral arteries that do not result in cerebral infarction; I67- Other cerebrovascular diseases; I68- Cerebrovascular disorders in diseases classified elsewhere; and I69- Sequelae of cerebrovascular diseases.^8^The population data necessary to calculate the indicators were obtained from the Brazilian Institute of Geography and Statistics (IBGE).^9^

### To obtain the rates the following equations were used:

a) Annual mortality rate: number of deaths due to CBVD in the local and year /local population and year X 100,000 inhabitants;

b) Mortality rate for the period (1996-2015): mean number of deaths from CBVD of the time series (1996-2015)/population median of the time series (population mean of 2005 and 2006) x 100.000 inhabitants.

Finally, the HDI was obtained from the human development atlas (http://atlasbrasil.org.br/2013/) and the SVI from the social vulnerability atlas (http://ivs.ipea.gov.br/index.php/pt/), based on the year 2010. It should be noted that the HDI and SVI data are only calculated in the census years.

### Standardization of mortality rates

In order to reduce the effects of the population-demographic structure, the crude rates were standardized by gender and age using the direct method, considering the Brazilian population in 2010 (census year) as the standard population and the following age groups: 0-4, 5 -9, 10-14, 15-19, 20-29, 30-39, 40-49, 50-59, 60-69, 70-79 and 80 or more.

### Statistical analysis

For the temporal analysis, the inflection point regression model (joinpoint regression model) was used. The model tests whether a line with multiple segments is more adequate to explain the temporal behavior of a data set when compared to a straight line or one with fewer segments. Therefore, the joinpoint allows identifying the trend of each indicator (whether stationary, increasing or decreasing), the points in time in which there is a change in this trend (joins), as well as the annual percentage variation (APC- Annual Percent Change) and the total period (AAPC- Average Annual Percent Change).^10^In the model configuration, the following parameters were adopted: minimum number of joins: zero; maximum number of joins: three; selection of the best model: Monte Carlo permutation test (n = 4499 permutations); error autocorrelation method: method based on date; confidence interval: 95% (95% CI); and significance level: 5%.

For the analysis of the association between social indicators and the standardized mortality rate, the multivariate regression model (OLS - Ordinary Least Square) was adopted.

For the analyses, the software Joinpoint Regression 4.5.0.1 (National Cancer Institute. USA), GeoDa 1.10.0.8 (University of Illinois at Urbana-Champaign, USA) and QGis 2.14.11 (Open Source Geospatial Foundation, USA) were used. The territorial meshes necessary for making the maps came from IBGE.

### Ethical aspects

This study used secondary data in the public domain, in which it is not possible to identify the subjects. For this reason, the local Research Ethics Committee approval has been waived.

## Results

Between 1996 and 2015, 1.850.811 deaths due to CBVD were recorded in Brazil, resulting in a mean of 92.540 cases/year. Of this total, 50.68% (n = 938.044) occurred in males and 77.80% (n = 1.440.170) in elderly people. The age group of 80 years or over was the only age group with a higher proportion of females than males. There was a high rate of low level of education: 39.94% (n = 739.233) were illiterate or had up to three years of schooling. In this variable, a high proportion of unknown fields was observed (38.29% / n = 708.685) ( Table 1 ).

**Table 1:** – Sociodemographic characterization of deaths due to Cerebrovascular diseases (CBVD), according to gender. Brazil, 1996-2015

Variables	Male n= 938044 (50.68%)		Female n= 912202 (49.29%)		Unknown n= 565 (0.03%)		Total of Deaths n= 1850811 (100%)	
	n	%	n	%	n	%	n	%
**Age range**								
0-4	1012	55.95	793	43.83	4	0.22	1809	1.00
5-9	565	53.25	496	46.75	0	0.00	1061	0.06
10-14	999	54.44	834	45.45	2	0.11	1835	0.10
15-19	1998	55.27	1616	44.70	1	0.03	3615	0.20
20-29	7158	52.66	6426	47.27	10	0.07	13594	0.73
30-39	21278	50.09	21186	49.87	17	0.04	42481	2.30
40-49	62652	50.98	60217	48.99	37	0.03	122906	6.64
50-59	124934	56.74	95185	43.23	65	0.03	220184	11.90
60-69	200551	57.92	145578	42.05	106	0.03	346235	18.71
70-79	268228	53.12	236627	46.85	135	0.03	504990	27.28
80 and over	246717	41.89	342104	58.09	124	0.02	588945	31.81
Unknown age	1952	61.85	1140	36.12	64	2.03	3156	0.17
**Years of study**								
Illiterate	162163	42.91	215672	57.07	90	0.02	377925	20.42
1-3 years	192038	53.15	169257	46.84	13	0.01	361308	19.52
4-7 years	126285	53.86	108156	46.13	11	0.01	234452	12.67
8-11 years	54461	54.17	46075	45.83	6	0.01	100542	5.43
12 years and more	29083	57.64	21369	42.35	4	0.01	50456	2.73
1-8 years *	4868	53.92	4158	46.06	2	0.02	9028	0.49
9-11 years *	4551	54.08	3860	45.87	4	0.05	8415	0.45
Unknown	364595	51.45	343655	48.49	435	0.06	708685	38.29
**Marital status**								
Single	163672	47.22	182828	52.75	87	0.03	346587	18.73
Married	499651	67.31	242565	32.67	143	0.02	742359	40.11
Widowed	152794	28.22	388484	71.76	124	0.02	541402	29.25
Divorced	40958	59.29	28116	40.70	6	0.01	69080	3.73
Other	12235	64.02	6875	35.97	1	0.01	19111	1.03
Unknown	68734	51.96	63334	47.88	204	0.16	132272	7.15

* Different grouping of years of study occurred because of changes in the death certificate in 2011.

When analyzing the time series, the mortality rate due to CBVD in the Brazilian population, considering both genders, showed a linear trend of reduction (APC -2.4%; 95% CI -2.7 to -2.0; p = 0.001), from 72.3/100,000 (1996) to 46.4/100,000 (2015). Similar behaviors were observed in the male population (APC -2.3%; 95% CI -2.6 to -1.9; p = 0.001) and in the female population (APC -2.4%; 95% CI -2.8 to -2.0; p = 0.001), of which rates decreased from 77.8 and 71.4/100.000 to 51.1 and 45.2/100.000, respectively ( Figure 1 ).

**Figure 1:** Spatial distribution and trend of mortality rates standardized by Cerebrovascular diseases (CBVD) in Brazilian states, all population and according to gender. Brazil, 1996-2015. APC: Annual Percent Change; hab.: inhabitant; nº: number; 95%CI: 95% Confidence Interval; CBVD: Cerebrovascular diseases.

Figure 1 shows that the spatial distribution of the mean rates is heterogeneous, being higher in the Southeast and South states and lower in the North states. The highest overall mean rates were observed in the states of Paraná (75/100,000) and Espírito Santo (71.3/100,000) and the lowest in the states of Rio Grande do Norte (40.9/100,000) and Bahia (48.0/100,000). The same scenario was observed for male mortality (Paraná with 83.4/100,000 and Espírito Santo with 79.8/100,000). In the female population, the highest rates were observed in Paraná (71.2/100,000) and Rio Grande do Sul (69.2/100,000) and the lowest in Rio Grande do Norte (40.7/100,000) and Bahia (49.1/100,000).

The trend in mortality rates was also analyzed considering the complete time series (1996-2015). The North region was the only one that showed a tendency towards an increase in mortality in the general population (APC 0.4%; 95% CI 0.1 to 0.8; p <0.001) and in the male population (APC 0.7%; 95% CI 0.3 to 1.1; p <0.001). The Midwest, Southeast and South regions showed a decreasing trend, both in the general population and in the male and female populations. The Southeast region showed the highest percentage of reduction in the time series (APC 3.8%) ( Table 2 ).

**Table 2:** – Percentage of Average Annual Variation (PAAV) of mortality rates standardized by Cerebrovascular diseases (CBVD), according to gender, in Brazil, regions and federation units. 1996-2015

Spatial Unit	Both genders			Male			Female		
	Rate^1^		AAPC (CI 95%) p value	Rate^1^		AAPC (CI 95%) p value	Rate^1^		AAPC (CI 95%) p value
	1996	2015		1996	2015		1996	2015	
North	50.5	58.6	0.4* (0.1 to 0.8); p<0.001	51.0	61.6	0.7* (0.3 to 1.1); p<0.001	52.6	58.6	0.1 (-0.3 to 0.5); p=0.6
RO	68.2	51.4	-1.8* (-2.2 to -1.8); p<0.001	65.9	53.3	1.6* (-2.3 to -1.0); p<0.001	74.2	51.5	-1.9*(-2.3 to -1.4); p<0.001
AC	57.4	63.1	-0.1 (-1.9 to 1.7); p=0.9	59.5	62.7	0.4 (-2.3 to 3.1); p=0.8	58.7	66.9	0.4 (-4.3 to 5.4); p=0.9
AM	49.7	56.1	0.6* (0.2 to 1.1); p<0.001	49.7	58.0	0.9* (0.2 to 1.5); p<0.001	52.7	57.3	0.4 (-0.1 to 1.0); p=0.1
RR	75.5	46.8	-2.2* (-3.0 to -1.3); p<0.001	92.0	50.7	-2.0* (-3.6 to -0.4); p<0.001	56.4	44.2	-2.3*(-3.3 to -1.3); p<0.001
PA	46.3	61.8	1.2 (-0.8 to 3.4); p=0.2	45.7	66.0	1.9 (-0.3 to 4.1); p=0.1	49.5	60.8	0.1 (-2.0 to 2.2); p=0.9
AP	79.5	49.2	-1.7 (-7.8 to 4.9); p=0.6	77.1	54.3	-0.8 (-4.8 to 3.5); p=0.7	86.2	46.6	-1.2 (-3.8 to 1.5); p=0.4
TO	43.4	59.3	1.9* (0.9 to 2.9); p<0.001	48.0	60.1	1.4 (-1.3 to 4.3); p=0.3	40.0	60.7	2.3* (0.7 to 3.9); p<0.001
Northeast	45.4	54.4	0.9 (-0.7 to 2.4); p=0.3	46.8	60.7	1.3 (-0.3 to 2.9); p=0.1	46.7	52.7	0.6 (-1.1 to 2.3); p=0.5
MA	29.0	68.2	4.6* (2.0 to 7.4); p<0.001	31.7	76.6	4.7* (2.5 to 7.0); p<0.001	27.2	64.8	4.3* (1.6 to 7.0); p<0.001
PI	33.3	76.9	3.9* (2.9 to 4.8); p<0.001	35.0	90.4	4.2* (2.9 to 5.5); p<0.001	33.0	70.3	4.0* (3.0 to 4.9); p<0.001
CE	42.0	55.1	1.3 (-0.2 to 2.8); p=0.1	43.5	62.6	1.7* (0.2 to 3.2); p<0.001	42.8	52.1	0.7 (-0.2 to 1.6); p=0.1
RN	33.0	38.0	0.9 (-0.1 to 1.8); p=0.1	34.4	43.7	1.3* (0.2 to 2.5); p<0.001	33.0	35.6	-0.4 (-0.8 to 1.5); p=0.5
PB	37.5	48.5	1.7* (0.4 to 3.0); p<0.001	39.0	52.2	1.9* (0.3 to 3.4); p<0.001	38.4	48.6	1.4 (-0.1 to 2.8); p=0.1
PE	64.8	58.0	-0.8 (-2.2 to 0.6); p=0.3	68.1	66.6	-0.4 (-1.0 to 0.2); p=0.2	65.9	55.2	-1.1 (-2.6 to 0.4); p=0.1
AL	55.5	69.3	0.8* (0.2 to 1.5); p<0.001	57.8	77.7	1.2* (0.6 to 1.8); p<0.001	57.1	66.9	0.5 (-0.5 to 1.5); p=0.3
SE	41.8	57.6	1.7* (1.0 to 2.3); p<0.001	45.7	64.7	1.9* (1.0 to 2.9); p<0.001	40.2	55.6	1.5* (0.5 to 2.5); p<0.001
BA	47.7	45.2	-0.0 (-0.6 to 0.5); p=0.9	47.4	47.5	0.2 (-0.3 to 0.8); p=0.4	51.0	46.2	-0.2 (-0.8 to 0.3); p=0.4
Midwest	69.5	46.3	-2.8* (-3.4 to -2.2); p<0.001	72.2	49.2	-2.7* (-3.3 to -2.2); p<0.001	69.1	46.2	-2.3 (-4.8 to 0.2); p=0.1
MS	76.9	52.9	-2.4* (-2.9 to -2.0); p<0.001	83.9	54.5	-2.4* (-3.0 to -1.8); p<0.001	73.1	54.5	-2.4* (-2.8 to 1.9); p<0.001
MT	65.7	44.2	-1.9* (-3.0 to -0.8); p<0.001	66.3	45.6	-2.1* (-3.5 to -0.6); p<0.001	67.6	44.8	-2.5*(-3.2 to -1.9); p<0.001
GO	64.2	46.2	-2.2* (-2.6 to -1.8); p<0.001	66.0	49.7	-1.6* (-2.7 to -0.4); p<0.001	65.1	45.6	-2.2 (-2.6 to -1.8); p<0.001
DF	81.3	41.6	-4.0* (-4.6 to -3.5); p<0.001	91.6	46.4	-4.0* (-4.6 to -3.4); p<0.001	77.2	40.5	-3.4 (-5.4 to -1.3); p<0.001
Southeast	86.1	41.4	-3.8* (-4.1 to -3.4); p<0.001	96.3	45.8	-3.8* (-4.2 to -3.5); p<0.001	82.3	40.2	-3.8*(-4.2 to -3.4); p<0.001
MG	74.1	39.3	-3.2* (-3.5 to -2.9); p<0.001	81.0	41.5	-3.3* (-3.7 to -3.0); p<0.001	71.5	39.5	-3.0*(-3.3 to -2.8); p<0.001
ES	98.4	46.7	-3.6* (-4.3 to -2.9); p<0.001	108.7	51.7	-3.5* (-4.0 to -3.1); p<0.001	94.2	45.1	-3.5*(-4.2 to -2.8); p<0.001
RJ	101.6	42.3	-4.5* (-5.3 to -3.7); p<0.001	113.8	47.9	-4.3* (-4.9 to -3.7); p<0.001	97.7	40.7	-4.5*(-5.8 to -3.2); p<0.001
SP	84.3	41.7	-3.8* (-4.2 to -3.4); p<0.001	95.8	46.7	-3.8* (-4.0 to -3.5); p<0.001	79.4	40.1	-3.8*(-4.0 to -3.5); p<0.001
South	91.0	45.9	-3.7* (-4.1 to -3.2); p<0.001	96.9	49.5	-3.7* (-4.1 to -3.2); p<0.001	91.3	45.6	-3.6*(-4.0 to -3.2); p<0.001
PR	98.9	49.9	-3.8* (-4.1 to -3.6); p<0.001	108.0	55.3	-3.8* (-4.1 to -3.5); p<0.001	95.5	48.0	-3.8*(-4.1 to -3.5); p<0.001
SC	89.1	37.7	-4.4* (-4.8 to -4.0); p<0.001	94.4	39.7	-3.9* (-5.9 to -2.0); p<0.001	89.5	38.2	-4.3*(-4.7 to -3.9); p<0.001
RS	86.1	46.5	-3.1* (-4.2 to -1.9); p<0.001	88.9	49.4	-3.0* (-3.6 to -2.3); p<0.001	89.3	47.0	-3.4*(-4.1 to -2.6); p<0.001

*Statistical significance(p<0.05);^*1*^– Mortality rate/100.000 inhabitants; AAPC: Average Annual Percent Change; RO: Rondônia; AC: Acre; AM: Amazonas; RR: Roraima; PA: Pará; AP: Amapá; TO: Tocantins; MA: Maranhão; PI: Piauí; CE: Ceará; RN: Rio Grande do Norte; PB: Paraíba; PE: Pernambuco; AL: Alagoas; SE: Sergipe; BA: Bahia; MG: Minas Gerais; ES: Espírito Santo; RJ: Rio de Janeiro; SP: São Paulo; PR: Paraná; SC: Santa Catarina; RS: Rio Grande do Sul; MS: Mato Grosso do Sul; MT: Mato Grosso; GO: Goiás; and DF: Distrito Federal.

In the stratified analysis by federation unit, 20 states showed significant trends, 7 showed increasing trends and 13 decreasing trends. All states in the Midwest, South and Southeast regions showed decreasing trends, with emphasis on Rio de Janeiro and Santa Catarina, with the highest reduction percentages. On the other hand, 5 of the 7 states with increasing trends are located in the northeast region (Maranhão, Piauí, Paraíba, Alagoas and Sergipe) and two in the north (Amazonas and Tocantins) ( Table 2 ).

Only the Federal District was classified with very high HDI (HDI 0.824). All states in the Northeast and five in the North had medium HDI (between 0.600 and 0.699), with Alagoas and Maranhão standing out with the lower values (HDI 0.631 and 0.639, respectively). In parallel, these same states in the North and Northeast regions had the highest values in the SVI, especially Maranhão with very high SVI (SVI 0.521). All eight states classified as showing high social vulnerability are located in the North (n = 4) and Northeast (n = 4) regions ( Figure 2 ).

**Figure 2:** – Spatial distribution of the Human Development Index (HDI) and the Social Vulnerability Index (SVI) in Brazilian states. Brazil, 2010.

The temporal regression model showed the states of the North and Northeast regions with the largest number of segments in the time series (joins), representing greater oscillation in rates over the years. The mortality rate in the Northeast showed four time segments: slight growth (1996-2003), stationary behavior (2003-2006), downward trend (2006-2010) and again a stationary behavior (2010-2015). Among the states in this region, only Bahia showed a linear behavior ( Table 3 ).

**Table 3:** – Percentage of annual variation in mortality rates standardized by Cerebrovascular diseases (CBVD), according to gender. Brazil. 1996-2015

Spatial unit	Both genders		Male		Female	
	Period	AAPC (95% CI) p value	Period	AAPC (CI 95%) p value	Period	AAPC (95% CI) p value
**North**	1996-2015	0.4* (0.0 to 0.8); p<0.001	1996-2015	0.7* (0.3 to 1.1); p<0.001	1996-2015	0.1 (-0.3 to 0.5); p=0.6
RO	1996-2015	-1.8*(-2.2 to -1.8) ; p<0.001	1996-2015	1.6* (-2.3 to -1.0); p<0.001	1996-2015	-1.9* (-2.3 to -1.4); p<0.001
AC	1996-1999	-16.2 (-30.7 to 1.4); p=0.1	1996-2002	-5.6 *(-9.4 to -1.7); p<0.001	1996-1999	-16.2 (-30.7 to 1.4); p=0.1
	1999-2006	9.9* (3.1 to 17.2); p<0.001	2002-2006	13.2* (0.5 to 27.5); p<0.001	1999-2006	9.9* (3.1 to 17.2); p<0.001
	2006-2011	-6.4 (-17 to 5.6); p=0.2	2006-2015	-0.8 (-2.9 to 1.4); p=0.4	2006-2011	-6.4 (-17.0 to 5.6); p=0.2
	2011-2015	7.1 (-5.0 to 20.8); p=0.2			2011-2015	7.1 (-5.0 to 30.8); p=0.2
AM	1996-2015	0.6* (0.2 to 1.1); p<0.001	1996-2015	0.9* (0.2 to 1.5); p<0.001	1996-2015	0.4 (-0.1 to 1.0); p=0.1
RR	1996-2015	-2.2* (-3.0 to -1.3); p<0.001	1996-2015	-2.0* (-3.6 to -0.4); p<0.001	1996-2015	-2.3* (-3.3 to -1.3); p<0.001
PA	1996-2004	-0.8 (-2.9 to 1.2); p=0.9	1996-1998	11.6 (-1.1 to 25.9); p=0.1	1996-2004	-0.8 (-2.9 to 1.2); p=0.4
	2004-2008	6.3 (-3.3 to 16.8); p=1.4	1998-2001	-4.2 (-15.1 to 8.2); p=0.4		
	2008-2015	-2.3 (-4.7 to 0.2); p=-2.0	2001-2008	4.3* (2.2 to 6.4); p<0.001	2004-2008	6.3 (-3.3 to 16.8); p=0.2
			2008-2015	-0.5 (-2.1 to 1.2); p=0.5	2008-2015	-2.3 (-4.7 to 0.2); p=0.1
AP	1996-2007	-5.8* (-8.8 to -2.7); p=0.6	1996-2002	1.9 (-4.3 to 8.4); p=0.5	1996-2007	-5.8* (-8.8 to -2.7); p<0.001
	2007-2015	5.5* (0.1 to 11.3); p<0.001	2002-2006	-11.1 (-26.1 to 7.0); p=0.2	2007-2015	5.5* (0.1 to 11.3); p<0.001
			2006-2015	2.4 (-1.0 to 5.9); p=0.2		
TO	1996-2003	11.6* (7.5 to 15.8); p<0.001	1996-2000	3.1 (-2.5 to 8.9); p=0.3	1996-2003	11.6* (7.5 to 15.8); p<0.001
	2003-2015	-2.8* (-4.4 to -1.1); p<0.001	2000-2003	15.3 (-3.1 to 37.3); p=0.1	2003-2015	-2.8* (-4.4 to -1.1); p<0.001
			2003-2015	-2.3* (-3.3 to -1.3); p<0.001		
**Northeast**	1996-2003	1.7* (0.5 to 2.9); p=0.3	1996-2003	2.1* (-0.9 to 3.3); p=0.1	1996-2003	1.4* (0.1 to 2.7); p<0.001
	2003-2006	7.4 (-1.5 to 17.2); p=1.9	2003-2006	6.6 (-2.3 to 16.4); p=0.1	2003-2006	8.3 (-1.5 to 19.1); p=0.1
	2006-2010	-4.5* (-8.5 to -0.2); p=0.3	2006-2010	-3.7 (-7.8 to 0.6); p=0.1	2006-2010	-5.1* (-9.5 to -0.5); p<0.001
	2010-2015	0.3 (-1.7 to 2.2); p=0.3	2010-2015	1.1 (-0.9 to 3.1); p=0.2	2010-2015	-0.4 (-2.5 to 1.8); p=0.7
MA	1996-2006	4.5* (2.2 to 6.9); p<0.001	1996-2000	0.1 (-7.1 to 7.8); p=1.0	1996-2003	4.5* (2.2 to 6.9); p<0.001
	2003-2006	18.3 (-0.2 to 40.2); p=0.1	2000-2007	13.4* (8.9 to 18.0); p<0.001	2003-2006	18.3 (-0.2 to 40.2); p=0.1
	2006-2015	-0.2 (-1.7 to 1.4); p=0.8	2007-2015	-0.1 (-2.6 to 2.6); p=1.0	2006-2015	-0.2 (-1.7 to 1.4); p=0.8
PI	1996-2006	8.9* (7.6 to 10.3); p<0.001	1996-2007	8.4* (6.8 to 10.1); p<0.001	1996-2006	8.9* (7.6 to 10.3); p<0.001
	2006-2015	-1.3 (-2.7 to 0.2); p=0.1	2007-2015	-1.4 (-3.7 to 1.1); p=0.2	2006-2015	-1.3 (-2.7 to 0.2); p=0.1
CE	1996-2007	2.8* (1.7 to 3.9); p<0.001	1996-1998	11.5 (-2.2 to 27.0); p=0.1	1996-2007	2.8* (1.7 to 3.9); p<0.001
	2007-2015	-2.2* (-3.9 to -0.4); p<0.001	1998-2008	2.0* (0.8 to 3.2); p<0.001	2007-2015	-2.2* (-3.9 to -0.4); p<0.001
			2008-2015	-1.2 (-2.9 to 0.5); p=0.1		
RN	1996-2009	2.4* (1.4 to 3.5); p<0.001	1996-2008	4.2* (2.9 to 5.4); p<0.001	1996-2009	2.4* (1.4 to 3.5); p<0.001
	2009-2015	-3.9* (-7.0 to -0.6); p<0.001	2008-2015	-3.3* (-5.9 to -0.6); p<0.001	2009-2015	-3.9* (-7.0 to -0.6); p<0.001
PB	1996-1998	-11.8*(-22.1 to 0.0); p<0.001	1996-1999	-5.5 (-12.3 to 2.3); p=0.1	1996-1998	-11.8* (-22.1 to 0.0); p<0.001
	1998-2007	8.9* (7.4 to 10.4); p<0.001	1999-2007	10.6* (8.3 to 13.0); p<0.001	1998-2007	8.9* (7.4 to 10.4); p<0.001
	2007-2015	-3.1* (-4.4 to -1.8); p<0.001	2007-2015	-3.5* (-5.1 to -1.8); p<0.001	2007-2015	-3.1* (-4.4 to -1.8); p<0.001
PE	1996-1998	5.1 (-2.8 to 13.7); p=0.2	1996-2006	1.1* (0.3 to 2.0); p<0.001	1996-1998	5.1 (-2.8 to 13.7); p=0.2
	1998-2001	-6.0 (-13.1 to 1.7); p=0.1	2006-2015	-2.0* (-3.0 to -1.1); p<0.001	1998-2001	-6.0 (-13.1 to 1.7); p=0.1
	2001-2005	4.9* (0.9 to 9.1); p<0.001			2001-2005	4.9* (0.9 to 9.1); p<0.001
	2005-2015	-3.2* (-3.8 to -2.6); p<0.001			2005-2015	-3.2* (-3.8 to -2.6); p<0.001
AL	1996-2007	2.4* (1.2 to 3.7); p<0.001	1996-2007	3.2* (2.4 to 4.0); p<0.001	1996-2007	2.4* (1.2 to 3.7); p<0.001
	2007-2015	-2.1 (-4.0 to -0.1); p<0.001	2007-2015	-1.5* (-2.7 to -0.3); p<0.001	2007-2015	-2.1* (-4.0 to -0.1); p<0.001
SE	1996-2005	5.7* (4.0 to 7.5); p<0.001	1996-2005	5.8* (4.1 to 7.4); p<0.001	1996-2005	5.7* (4.0 to 7.5); p<0.001
	2005-2015	-2.1* (-3.5 to -0.7); p<0.001	2005-2015	-1.4* (-2.7 to -0.1); p<0.001	2005-2015	-2.1* (-3.5 to -0.7); p<0.001
BA	1996-2015	-0.0 (-0.6 to 0.5); p=0.9	1996-2015	0.2 (-0.3 to 0.8); p=0.4	1996-2015	-0.2 (-0.8 to 0.3); p=0.4
**Midwest**	1996-2015	-2.8* (-3.4 to -2.2); p<0.001	1996-2015	-2.7* (-3.3 to -2.2); p<0.001	1996-2005	-0.5 (-2.0 to 1.0); p=0.5
					2005-2008	-0.9 (-23.1 to 7.7); p=0.2
					2008-2015	-1.6 (-3.8 to 0.6); p=0.1
MS	1996-2015	-2.4* (-2.9 to -2.0); p<0.001	1996-2015	-2.4* (-3.0 to -1.8); p<0.001	1996-2015	-2.4* (-2.8 to 1.9); p<0.001
MT	1996-2015	-1.9* (-3.0 to -0.8); p<0.001	1996-1998	9.7 (-3.5 to 24.6); p=0.1	1996-2015	-2.5* (-3.2 to -1.9); p<0.001
			1998-2010	-2.3* (-3.1 to -1.4); p<0.001		
			2010-2015	-5.9* (-8.6 to -3.2); p<0.001		
GO	1996-2015	-2.2* (-2.6 to -1.8); p<0.001	1996-1999	2.9 (-3.0 to 9.1); p<0.001	1996-2015	-2.2 (-2.6 to -1.8); p<0.001
			1999-2007	-3.8* (-5.3 to -2.3); p<0.001		
			2007-2015	-0.9 (-2.1 to 0.4); p=0.2		
DF	1996-1998	5.7 (-14.4 to 30.6); p=0.6	1996-2015	-4.0* (-4.6 to -3.4); p<0.001	1996-1998	5.7 (-14.4 to 30.6); p=0.6
	1998-2015	-4.4* (-5.1 to -3.7); p<0.001			1998-2015	-4.4* (-5.1 to -3.7); p<0.001
Southeast	1996-2015	-3.8* (-4.1 to -3.4); p<0.001	1996-2015	-3.8* (-4.2 to -3.5); p<0.001	1996-2015	-3.8* (-4.2 to -3.4); p<0.001
MG	1996-2009	-2.6* (-3.2 to -1.9); p<0.001	1996-2015	-3.3* (-3.7 to -3.0); p<0.001	1996-2015	-3.0* (-3.3 to -2.8); p<0.001
	2009-2015	-5.5* (-7.4 to -3.5); p<0.001				
ES	1996-2015	-3.6 (-4.3 to -2.9); p<0.001	1996-2015	-3.5* (-4.0 to -3.1); p<0.001	1996-2009	-2.6* (-3.2 to -1.6); p<0.001
					2009-2015	-5.5* (-7.4 to -3.5); p<0.001
RJ	1996-2005	-5.1* (-5.9 to -4.4); p<0.001	1996-2010	-3.9* (-4.4 to -3.5); p<0.001	1996-2005	-5.1* (-5.9 to -4.4); p<0.001
	2005-2008	-0.6 (-8.9 to 8.4); p=0.9	2010-2015	-5.4* (-7.4 to -3.3); p<0.001	2005-2008	-0.6* (-8.9 to 8.4); p<0.001
	2008-2015	-5.4* (-6.5 to -4.3); p<0.001			2008-2015	-5.4* (-6.5 to -4.3); p<0.001
SP	1996-2015	-3.8* (-4.2 to -3.4); p<0.001	1996-2015	-3.8* (-4.0 to -3.5); p<0.001	1996-2015	-3.8* (-4.0 to -3.5); p<0.001
South	1996-2015	-3.7* (-4.1 to -3.2); p<0.001	1996-2015	-3.7* (-4.1 to -3.2); p<0.001	1996-2015	-3.6* (-4.0 to -3.2); p<0.001
PR	1996-2015	-3.8* (-4.1 to -3.6); p<0.001	1996-2015	-3.8* (-4.1 to -3.5); p<0.001	1996-2015	-3.8* (-4.1 to -3.5); p<0.001
SC	1996-2015	-4.4* (-4.8 to -4.0); p<0.001	1996-1998	5.2 (-9.2 to 21.9); p=0.5	1996-2015	-4.3* (-4.7 to -3.9); p<0.001
			1998-2002	-8.3*(-14.8 to -1.3); p<0.001		
			2002-2015	-3.9* (-4.7 to -3.2); p<0.001		
RS	1996-2012	-3.0* (-3.0 to -3.4); p<0.001	1996-1998	4.7 (-0.0 to 9.6); p<0.001	1996-2012	-3.0* (-3.4 to -2.6); p<0.001
	2012-2015	-5.4* (-10.0 to -0.6); p<0.001	1998-2006	-3.9* (-4.5 to -3.3); p<0.001		
			2006-2010	-1.6 (-3.8 to 0.7); p=0.1	2012-2015	-5.4* (-10.0 to -0.6); p<0.001
			2010-2015	-5.5* (-6.4 to -4.5); p<0.001		

*Statistical significance(p<0.05; AAPC: Average Annual Percent Change; RO: Rondônia; AC: Acre; AM: Amazonas; RR: Roraima; PA: Pará; AP: Amapá; TO: Tocantins; MA: Maranhão; PI: Piauí; CE: Ceará; RN: Rio Grande do Norte; PB: Paraíba; PE: Pernambuco; AL: Alagoas; SE: Sergipe; BA: Bahia; MG: Minas Gerais; ES: Espírito Santo; RJ: Rio de Janeiro; SP: São Paulo; PR: Paraná; SC: Santa Catarina; RS: Rio Grande do Sul; MS: Mato Grosso do Sul; MT: Mato Grosso; GO: Goiás; and DF: Distrito Federal.

Finally, the regression model showed a positive association between the mean mortality rate and the Municipal Human Development Index (p = 0.046), with the income dimension (p = 0.029), and a negative association with the general SVI (p = 0.026) and also in two dimensions: human capital (p = 0.046) and income and work (p = 0.018) ( Table 4 ).

**Table 4:** – Regression model (OLS, Ordinary least square) between the mortality rate due to Cerebrovascular diseases (CBVD) and the Human Development Index (HDI) and Social Vulnerability Index (SVI). Brazil, 1996-2015

Variable	Coefficient	t Statistics	p value
**Municipal Human Development Index (MHDI)**	61.588	2.091	0.046*
MHDI Longevity	90.265	1.866	0.073
MHDI Education	47.075	1.861	0.074
MHDI Income	56.476	2.301	0.029*
**Social Vulnerability Index (SVI)**	-40.802	-2.353	0.026*
SVI Urban infrastructure	-15.998	-1.110	0.277
SVI Human capital	-31.883	-2.092	0.046*
SVI Income and work	-35.322	-2.528	0.018*

* significant association

## Discussion

Brazil has one of the highest mortality rates due to CBVD among the countries of Latin America and much higher than those observed in developed nations.^11^However, a temporal decline behavior has been observed over the last decades,^1^in the male and female populations, corroborating the national and international literature.^12 - 15^

Several authors have emphasized that such reduction in mortality can be explained by the expansion of access to health services and the adoption of prevention strategies.^14 , 15^In Brazil, the implementation of primary health care (PHC) stands out. The Family Health Strategy (FHS) develops actions to control risk factors, such as encouraging physical activity and adopting healthy eating habits, smoking control programs, diagnosis and systematic monitoring of chronic conditions (hypertension and diabetes, for example) and access to pharmaceutical assistance.^16 , 17^Between 1998 and 2017 there was a significant increase in the number of family health teams, going from approximately 2.000 to 41.000, reaching a coverage of 70% of the Brazilian population, which corresponds to approximately 143 million people.^17 , 18^Studies showed an association between the expansion of primary care and the reduction of mortality from diseases such as acute myocardial infarction and cerebrovascular diseases.^19^

In addition to PHC, Brazil has also advanced in the care of patients with CBVD. In 1997, the first stroke unit was implemented in Brazil, located in Joinville/SC. Based on this experience, in 2008, the Ministry of Health started the organization of the national stroke care network, resulting in Ordinance number 665/2012, with the purpose of implementing stroke referral services across the country.^20 , 21^

Another important action is the Strategic Action Plan for Confronting Chronic Noncommunicable Diseases (NCDs). Implemented in 2011 by the Ministry of Health, the plan established a set of goals for the country, such as the reduction of premature mortality due to NCDs, the prevalence of smoking and alcohol consumption in the population, an increase in the prevalence of physical activity and fruit consumption and containment of obesity increase.^22^

In the regional analysis, we found a heterogeneous behavior in the pattern of mortality from CBVD in the country, corroborating other studies.^4 , 23^Mortality rates were higher in the Southeast and South, but with a significant decreasing trend. In contrast, the North and Northeast regions had the lowest rates, but with a significant increasing trend over the historical series. This heterogeneous epidemiological-spatial context is the result of social, economic, demographic and epidemiological differences between the regions. Because of this, the results must be analyzed from the perspective of three dimensions: i) demographic and epidemiological transition; ii) social determinants of health and iii) quality of information systems.

Since the 1940s, Brazil has going through important demographic changes: a reduction in the overall mortality rate and a decline in birth rates have resulted in major changes in the demographic regime and in the age structure of the population, with a significant increase in the number of elderly individuals.^24^In 2000, this population was just over 14.2 million, increasing to 19.6 million in 2010, and is expected to reach 41.5 million by 2030,^25^with a greater concentration in the Southeast and South regions. The impact of the population aging process on the pattern of morbidity and mortality is significant, since it implies an increase in chronic diseases,^26^among which CBVD stand out. In our study, 77.8% of deaths occurred among the elderly.

Studies indicate that the risk of mortality from CBVD in the elderly population is substantially higher than in other age groups. One reason is the accumulation of risk factors, such as hypertension, diabetes, dyslipidemia, alcoholism, smoking and inappropriate eating habits.^27 , 28^In Brazil, for example, the prevalence of hypertension can affect 68% of the elderly population.^29^

Furthermore, the demographic transition process occurs concurrently with a second transition, the epidemiological one, characterized by changes in the population’s illness profile.^30^In the last decades, there has been a decline in infectious and parasitic diseases and an increase in the occurrence of chronic-degenerative diseases, many of which increase the risk of mortality from CBVD.^27^The North and Northeast regions are the most exposed to social vulnerability and show the lowest human development index, resulting in higher mortality from diseases related to an unfavorable social context and less from CBVD. In contrast to what was observed in the most developed regions of the country (Southeast and South). In this sense, the higher rates observed in the more developed states reflect social differences, and, consequently, greater participation of chronic conditions in the mortality profile. On the other hand, more vulnerable regions may have lower rates due to the persistence of mortality due to diseases related to poverty.^32^

The two transitions do not occur homogeneously in Brazil, with a mismatch between regions.^31^This phenomenon explains, in parts, the differences between Brazilian regions regarding CBVD mortality. This scenario justifies the positive association between CBVD mortality and human development and its negative association with social vulnerability, which represents the influence of the epidemiological and social context on the population’s mortality profile.

However, the isolated analysis of the rates is not enough to understand the epidemiological dynamics of CBVD. In the North and Northeast regions, in general, the rates showed a temporal pattern of growth and, in the Southeast and South regions, a decline was observed. These findings reflect the influence of social determinants of health on the pattern of mortality from CBVD. Socioeconomic conditions, including human development, income status and educational situation, have a significant influence on the risk of an individual dying from this group of diseases.^4 , 6 , 33 - 35^

A recent study of the Global Burden of Disease showed that Brazilian states located at the lower tertile of the Social Development Index showed lower reductions in mortality rates, when compared to states located in the upper tertile of development. The lower tertile comprised only states in the North and Northeast regions.^4^It is suggested that better living conditions have a dual influence on the mortality trend: i) reduce risk factors for the occurrence of disease events; and ii) contribute to patient survival when such events occur, reducing the chance of death.

Finally, it is necessary to reflect on the quality of the mortality records. It is a challenge to adequately monitor the population’s health conditions. The inadequate filling out of the death certificates, resulting in a high number of garbage codes, the difficulties in carrying out epidemiological investigations with undefined recorded deaths and the lack of trained human resources to act in the death surveillance services are common problems evidenced throughout the country, although the North and Northeast regions are the most affected by the problem.^36 , 37^The dubious quality of the information is an important limitation of this study.

Between 1996 and 2005, the percentage of deaths with undefined causes in these regions was higher than 20%, being even higher in the elderly population when compared to other age groups.^38^In this sense, mortality rates in the North and Northeast, for example, may be higher than the ones we disclosed in this study. On the other hand, it is necessary to highlight that in recent years, important advances in the quality of information have been observed in these regions.^13^

## Conclusion

Mortality from CBVD in Brazil shows an irregular epidemiological behavior across the regions. The highest rates were observed in states with a better human development index and less social vulnerability, but with a decreasing trend over the time series. On the other hand, in less developed states and with greater vulnerability, the rates were lower, but with an upward trend. In this sense, we recommend that public policies should be developed considering the regional/local context.
